# Performance of Machine Learning and Texture Analysis for Predicting Response to Neoadjuvant Chemoradiotherapy in Locally Advanced Rectal Cancer with 3T MRI

**DOI:** 10.3390/tomography8040173

**Published:** 2022-08-19

**Authors:** Davide Bellini, Iacopo Carbone, Marco Rengo, Simone Vicini, Nicola Panvini, Damiano Caruso, Elsa Iannicelli, Vincenzo Tombolini, Andrea Laghi

**Affiliations:** 1 Department of Radiological Sciences, Oncology and Pathology, “Sapienza” University of Rome—I.C.O.T. Hospital, Via Franco Faggiana, 1668, 04100 Latina, Italy; 2 Department of Surgical and Medical Sciences and Translational Medicine, “Sapienza” University of Rome—Diagnostic Imaging Unit, Sant′Andrea University Hospital, Via di Grottarossa 1035, 00189 Rome, Italy; 3 Department of Radiotherapy, Policlinico Umberto I, “Sapienza” University of Rome, 00161 Rome, Italy

**Keywords:** rectal neoplasms, neoadjuvant chemoradiotherapy, magnetic resonance imaging, texture analysis, artificial intelligence, machine learning

## Abstract

**Background**: To evaluate the diagnostic performance of a Machine Learning (ML) algorithm based on Texture Analysis (TA) parameters in the prediction of Pathological Complete Response (pCR) to Neoadjuvant Chemoradiotherapy (nChRT) in Locally Advanced Rectal Cancer (LARC) patients. **Methods:** LARC patients were prospectively enrolled to undergo pre- and post-nChRT 3T MRI for initial loco-regional staging. TA was performed on axial T2-Weighted Images (T2-WI) to extract specific parameters, including skewness, kurtosis, entropy, and mean of positive pixels. For the assessment of TA parameter diagnostic performance, all patients underwent complete surgical resection, which served as a reference standard. ROC curve analysis was carried out to determine the discriminatory accuracy of each quantitative TA parameter to predict pCR. A ML-based decisional tree was implemented combining all TA parameters in order to improve diagnostic accuracy. **Results:** Forty patients were considered for final study population. Entropy, kurtosis and MPP showed statistically significant differences before and after nChRT in patients with pCR; in particular, when patients with Pathological Partial Response (pPR) and/or Pathological Non-Response (pNR) were considered, entropy and skewness showed significant differences before and after nChRT (all *p* < 0.05). In terms of absolute value changes, pre- and post-nChRT entropy, and kurtosis showed significant differences (0.31 ± 0.35, in pCR, −0.02 ± 1.28 in pPR/pNR, (*p* = 0.04); 1.87 ± 2.19, in pCR, −0.06 ± 3.78 in pPR/pNR (*p* = 0.0005); 107.91 ± 274.40, in pCR, −28.33 ± 202.91 in pPR/pNR, (*p* = 0.004), respectively). According to ROC curve analysis, pre-treatment kurtosis with an optimal cut-off value of ≤3.29 was defined as the best discriminative parameter, resulting in a sensitivity and specificity in predicting pCR of 81.5% and 61.5%, respectively. **Conclusions:** TA parameters extracted from T2-WI MRI images could play a key role as imaging biomarkers in the prediction of response to nChRT in LARC patients. ML algorithms can be used to efficiently combine all TA parameters in order to improve diagnostic accuracy.

## 1. Introduction

Neoadjuvant Chemoradiotherapy (nChRT) followed by radical surgery accomplished by Total Mesorectal Excision (TME) represents the standard of treatment in Locally Advanced Rectal Cancer (LARC), having been shown to improve local tumor control.

However, once nChRT is completed, oncologists and surgeons usually have to deal with three different clinical scenarios. The first one is constituted by patients who completely respond to nChRT; modern nChRT schemes have proven able to obtain Pathological Complete Response (pCR) in around 18–25% of patients [1,2], meaning that surgery might be considered an overtreatment in this particular sub-group. Moreover, new evidence shows that a “watch-and-wait” approach might represent a feasible alternative instead of surgery [3,4], with several advantages, including organ sparing (preserving continence and bowel, sexual and urinary functions) and the absence of post-operative morbidity. The second scenario is accounted for by patients who do not respond to nChRT and might have obtained benefit from surgical treatment alone, thereby in vain exposed to the long-term toxicity of chemotherapy drugs and radiotherapy. Lastly, there are patients who show partial response to nChRT and might have undergone alternative therapeutical strategies [5,6]. In this setting, the early identification of these sub-groups of patients is of upmost importance in order to select patients for specific individual therapy.

During the last few years, translational research in oncologic imaging has produced a wide spectrum of potential biomarkers that could be useful for diagnosis, treatment, and prognosis, especially in the case of LARC. Among all imaging biomarkers available in rectal cancer, to the best of our knowledge, none have shown clinically relevant prognostic value in the prediction of tumoral response to nChRT.

Recently, Texture Analysis (TA) has been introduced as a novel quantitative imaging biomarker into research and clinical practice. TA is able to quantitatively assess heterogeneity of tissues, considered an important feature of malignancy associated with intrinsic biological tumoral behavior. Evidence suggests that TA can be easily integrated into morphological Magnetic Resonance Imaging (MRI) and Computed Tomography (CT) imaging to evaluate response during and after cancer treatment, playing at the same time an important role as prognostic factor [7,8,9,10]. Moreover, according to several cases reported in the literature, TA features obtained from LARC imaging studies may predict the outcome before surgery [8].

Another emerging field in medicine and radiology is Machine Learning (ML). ML is a subfield of Artificial Intelligence (AI), primarily focused on the development of predictive algorithms through unbiased identification of patterns among large datasets, not being explicitly set for a particular task. One of the most interesting potential employments of these models is its capacity to magnify data obtaining more meaningful information from radiological imaging datasets, especially if large and complex such as those obtained with TA.

With this in mind, the primary goal of the present study was to evaluate whether TA parameters obtained from MRI images could be used as imaging biomarkers able to discriminate a priori patients who would achieve a complete response after nChRT, using both conventional analysis and ML algorithms.

## 2. Material and Methods

This was a prospective, single-center study. Our institutional ethics committee approved this study, and written informed consent was obtained from all patients.

### 2.1. Study Population

We prospectively enrolled consecutive patients affected by LARC between July 2020 and January 2021, all of whom had histologically confirmed adenocarcinoma sub-type and tumor stages II (cT3-4, N0, M0) and III (cT1-4, N+, M0), according to the International Union Against Cancer classification [11].

Patients with the following criteria were excluded from further analysis: contraindication to MRI examination (e.g., pacemaker, intra-ocular metal foreign bodies, etc.); MRI protocol not fully acquired; incomplete histopathological data; contraindications to nChRT or surgical treatment, or suspension of nChRT before surgery; coexistence of other known tumors, or previous pelvic radiation treatment; legal incapacity or restricted legal capacity.

### 2.2. Protocol Timeline

**Figure 1:**
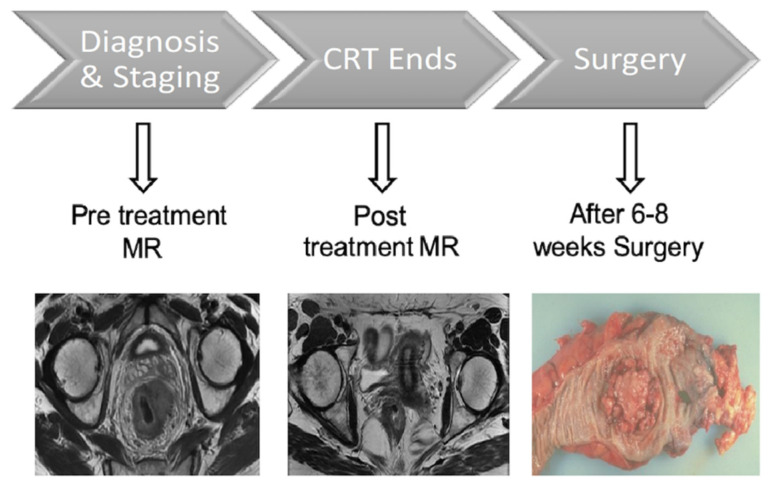
Study protocol timeline. CRT: Neoadjuvant Chemoradiotherapy.

The study, part of a larger study founded by AIRC (Associazione Italiana per la Ricerca sul Cancro), comprised two consecutive phases in which two MRI examinations were performed. The first MRI study included initial evaluation of local tumor status after histological confirmation. The second MRI study was performed at the end of the nChRT treatment scheme. Patients underwent TME 6–8 weeks after the end of nChRT, and histopathological assessment of the gross specimen was performed (Figure 1).

### 2.3. MRI Examination Acquisition Technique

MRI examinations were performed using a 3T scanner (Discovery MR750; General Electric) following a standard imaging protocol for rectal cancer evaluation as described in a previous study [12]. For the specific purpose of the study, high-resolution T2 weighted-imaging (T2-WI) fast recovery fast-spin echo (2D) sequences (repetition time, 2086–4172 milliseconds; echo time, 11.4–122.3 milliseconds; Nex, 2; slice thickness, 4 mm; matrix, 512 × 512) acquired on dedicated axial oblique planes orthogonal to the long axis of the rectum were analyzed.

### 2.4. Neoadjuvant Chemoradiotherapy Scheme

For radiation therapy, a total dose of 45 Gy was delivered to the whole pelvis with a 3-D conformational multiple field technique (fractioned in 25 daily administrations of 1.8 Gy per day for 5 weeks); an additional dose of 5.4 to 9 Gy was delivered to the tumor volume (fractioned in 3–5 per day administration of 1.8 Gy each), with 6 to 15 MV energy photons.

Chemotherapy protocol consisted of oxaliplatin and 5-fluoruracile intravenous infusion: oxaliplatin was administered the first day of each radiation therapy weekly session (2-h infusion, 50 mg/m^2^), while 5-fluroracile infusion continued for a total of 5 days (200 mg/m^2^/d).

### 2.5. Surgical Technique and Histopathological Assessment

Colorectal surgeons, with at least 10 years of experience, performed a standardized TME in all patients [13].

Expert gastro-intestinal pathologists performed the histopathology assessment by evaluating the basic histopathology of the primary biopsied tumor (type and grade of the lesion) before nChRT, as well as histopathological assessment of resected tumor specimens. In order to allow a comparison as probable as possible with MRI analysis, the intestinal resected segment containing the tumor was orthogonally sectioned with respect to the major axis, obtaining a macro-section with a thickness of 2–3 mm. Due to this technique, it was possible to maintain the original antero-posterior and left–right orientation of each specimen, making it easier to localize the pathological area seen on MRI. Tumor type and grade and the T and N stage were evaluated for all the specimens (according to 8th edition of the American Joint Commission on Cancer), together with the examination of the surgical margins.

Pathological response was assessed evaluating the Tumor Regression Grade (TRG) performing a complete analysis of all specimens. TRG evaluation was based on the amount of inflammatory tissue and fibrosis versus the portion of residual viable tumor, in accordance with the following grading scale: 0—no regression; 1—minor regression (with fibrosis ≤ 25% of the dominant tumor mass); 2—moderate regression (with fibrosis from 26% to 50% of the dominant tumor mass); 3—good regression (fibrosis > 50% of the dominant tumor mass); 4—complete regression (only fibrotic tissue, without viable tumor) [14]. According to TRG pathological response, patients were subdivided into three groups: Pathological Complete Response (pCR), with TRG of 4; Pathological Partial Response (pPR), with TRG of 2 and 3; Pathological Non-Response (pNR), with TRG of 0 and 1.

### 2.6. Texture Analysis

TexRAD (TexRAD Ltd., London, UK), a proprietary software algorithm, was used to extract and evaluate TA features obtained from LARC MRI images.

**Figure 2:**
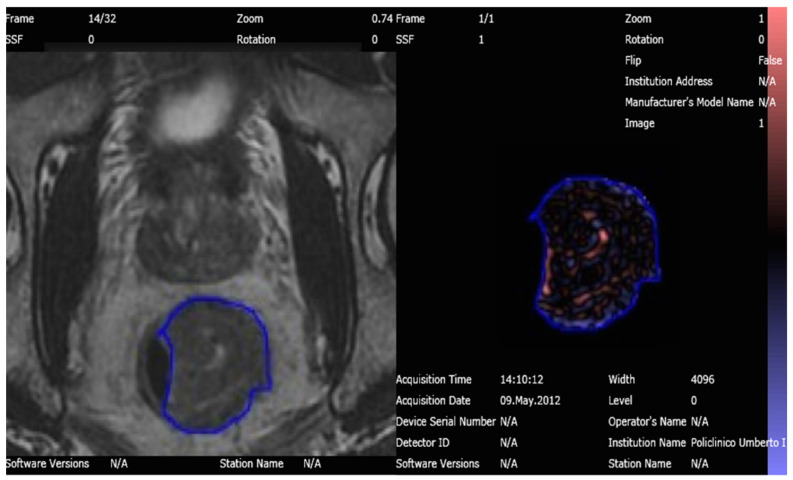
T2 Weighted-Image on 3T MRI of the rectal tumor before nChRT segmented and analyzed with Texture Analysis software.

An abdominal imaging-experienced radiologist, with 5 years of experience in TA, manually segmented a Region of Interest (ROI) around the largest tumor area appreciable on axial oblique T2-WI, avoiding cystic or necrotic regions, in both MRI examinations obtained before and after the treatment, respectively; the radiologist was not aware of the histological results and clinical data of the patients (Figure 2). The ROIs extracted were then analyzed for TA using the image histogram (first order) statistical method that refers to the frequency of the intensity of pixels.

An in-plane filtration step was adopted by means of a Laplacian of Gaussian spatial band-pass filter, producing a series of derived images highlighting features at different anatomic spatial scales, ranging from fine to coarse texture. The scale was defined by altering the spatial scale filter (SF) value between 0 and 2 in order to extract MRI intensity features of different sizes varying between 0 and 2 mm. Since analyses performed on images of different patients might require different values for the acquisition parameters, brightness and contrast of individual ROIs often differ. Accordingly, image normalization was performed in order to reduce image heterogeneity among the different patients as well as standardizing individual ROIs as much as possible [15]. Heterogeneity within the ROI was quantified with and without image filtration using the following histogram parameters: kurtosis, skewness, entropy, and mean value of positive pixels (MPP) [8,10,16]. As described by Miles and colleagues [17], entropy quantifies the irregularity of gray-level distribution, kurtosis expresses peakedness and tailedness of the histogram, while skewness identifies pixel distribution asymmetry and MPP expresses the average brightness of positive pixel values within the image.

Moreover, the absolute value changes after nChRT in terms of TA parameters were also computed as the difference between pre-treatment and post-treatment TA values.

For data analysis purposes, pCR, pPR and pNR patients were further grouped into pCR group and pPR/pNR group, and TA feature values were assessed and compared between these two groups.

### 2.7. Artificial Intelligence Analysis

A dedicated online software (WEKA^®^) [18] was used for AI analysis. Weka is a collection of ML algorithms for data mining tasks. It contains tools for data preparation, classification, regression, clustering, association rules mining, and visualization. Moreover, it allows for the construction of decision tree classifiers with the aim of correlating different parameters to define the best sequences for the proposed tasks: in our case, the ability to detect the “future complete responders” during the first MRI examination. We evaluated the impact of ML algorithms using TA features obtained from LARC MRI images combining all TA parameters and all filters applied. Despite that model testing or validation was not performed, for the scope of this work, WEKA outputs allowed us to over-cross validation for this task [19].

### 2.8. Statistical Analysis

Categorical variables were summarized as counts with percentages, and continuous variables were summarized as means with standard deviation (SD) and ranges. Categorical variables were compared between groups using the chi-squared test, and continuous variables were compared among the three groups using the Kruskal–Wallis test. Non-parametric Mann–Whitney U test was used to compare TA parameters (kurtosis, skewness, entropy, MPP) and the response rate among pCR and pPR/pNR groups before and after nChRT. Moreover, each parameter was also compared between the different patient sub-groups.

We performed further analyses assessing absolute changes among different texture parameters in pCR and pPR/pNR patients before and nChRT.

To evaluate performance of TA parameters, a Receiver Operating Characteristic (ROC) curve analysis was performed to assess the discriminatory power of TA parameters to predict pCR by calculating the areas under the ROC curve (AUCs) and the corresponding *p* values. ROC curves were computed from mean values of all filters combined and from all filters independently. Pre-nChRT datasets were evaluated. Optimal cut-off values were calculated as the cutoff thresholds maximizing the Youden index J, where J = sensitivity + specificity − 1. Sensitivity and specificity were calculated for the determined optimal cut-off values.

Statistical analysis was carried out using MedCalc version 12.7.2 (MedCalc Software). *p* values of <0.05 were considered statistically significant.

## 3. Results

### 3.1. Study Population

**Figure 3:**
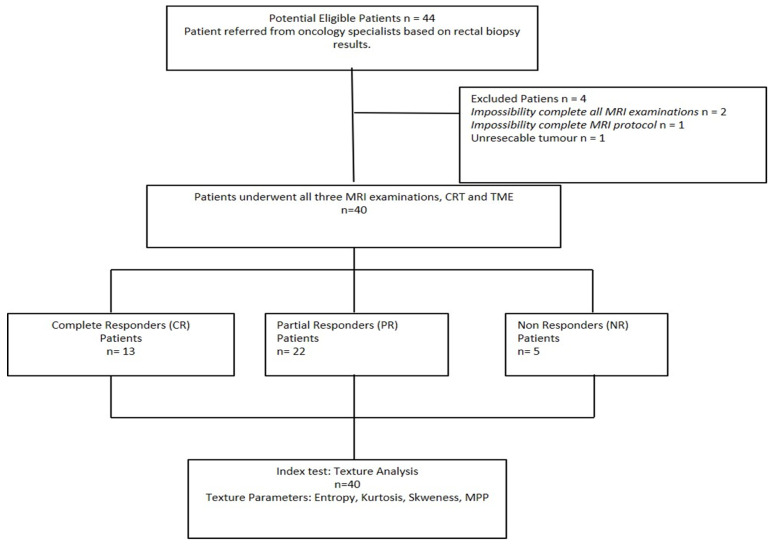
Study flow diagram of patient recruitment. CR: Complete Response; CRT: Neoadjuvant Chemoradiotherapy; MRI: Magnetic Resonance Imaging; NR: Non-Response; PR: Partial Response; TME: Total Mesorectal Excision.

The subjects’ accrual flow-chart is described in Figure 3. Four subjects were excluded from the study due to incomplete MRI protocol (*n* = 3), and unresectable lesion with impossibility to obtain a correct histopathologic assessment (*n* = 1).

The final study cohort thus consisted of 40 patients (mean age: 64 ± 9 years; age range: 39–82 years), including 24 men (mean age: 63 ± 9 years; age range: 39–82 years) and 16 women (mean age: 65 ± 10 years; age range, 47–81 years).

Pathological response to therapy was confirmed by means of histopathological analysis after TME for all patients. Thirteen patients showed pCR, twenty-two patients pPR, and five patients pNR.

Baseline demographic and clinical characteristics of patients included in the final study population are presented in Table 1.

### 3.2. Texture Analysis Values

**Figure 4:**
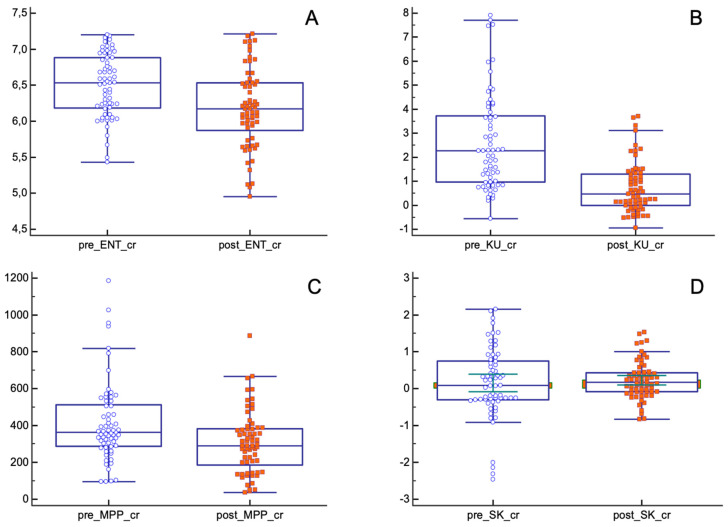
Differences in texture parameters before and after nChRT in pCR population. All differences are statistically significant except for skewness. (**A**) ENT: Entropy; (**B**) KU: Kurtosis; (**C**) MPP: Mean of Positive Pixels; (**D**) SK: Skewness.

In the pCR population, the mean values of entropy, kurtosis and MPP significantly decreased after nChRT (entropy (pre-nChRT: 6.49 ± 0.43, post-nChRT: 6.17 ± 0.54; *p* < 0.0001); kurtosis (pre-nChRT: 2.60 ± 2.01, post-nChRT: 0.72 ± 1.05; *p* < 0.0001); MPP (pre-nChRT: 414.24 ± 0219.26, post-nChRT: 306.33 ± 168.97; *p* = 0.0023)) (Figure 4).

Skewness showed similar results both before and after nChRT (pre-nChRT: 0.15 ± 0.96, post-nChRT: 0.22 ± 0.51; *p* = 0.53).

**Figure 5:**
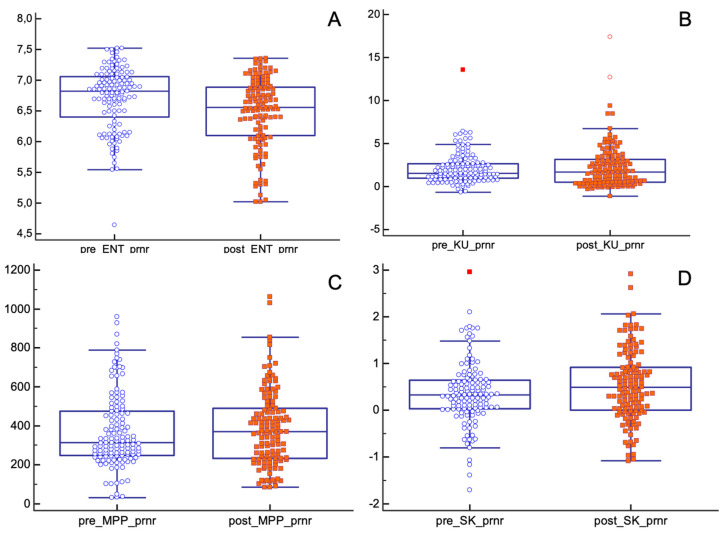
Differences in texture parameters before and after nChRT in pPR/pNR population. Values of entropy and skewness are statistically different before and after nChRT. (**A**) ENT: Entropy; (**B**) KU: Kurtosis; (**C**) MPP: Mean of Positive Pixels; (**D**) SK: Skewness.

As for the pPR/pNR group, only entropy and skewness showed statistically significant differences before and after nChRT (entropy (pre-nChRT: 6.70 ± 0.50, post-nChRT: 6.47 ± 0.57; *p* < 0.0001); skewness (pre-nChRT: 0.35 ± 0.67, post-nChRT: 0.53 ± 0.75; *p* = 0.029)) (Figure 5).

**Figure 6:**
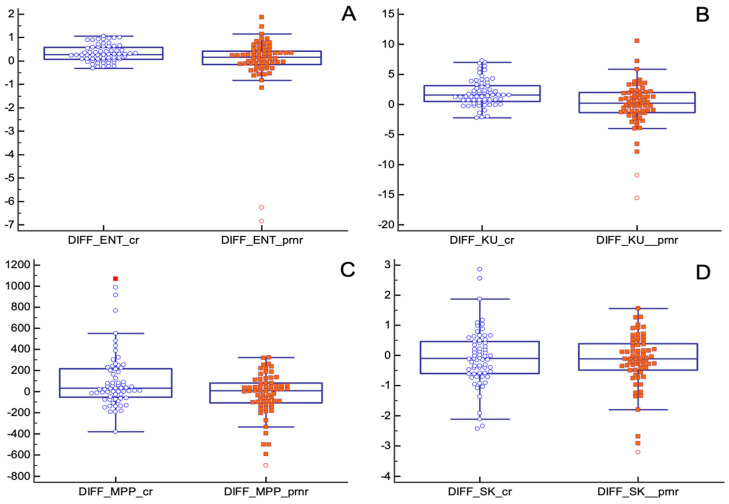
Absolute changes in texture parameters before and after nChRT in pCR and pPR/pNR groups. All differences are statistically significant except for skewness. (**A**) ENT: Entropy; (**B**) KU: Kurtosis; (**C**) MPP: Mean of Positive Pixels; (**D**) SK: Skewness.

Absolute changes in entropy, kurtosis and MPP before and after nChRT, comparing pCR and pPR/pNR groups, showed statistically significant results. TA parameters seemed to decrease after nChRT more in pCR patients compared to pPR/pNR patients (entropy (absolute reduction: 0.31 ± 0.35, in pCR group, −0.02 ± 1.28 in pPR/pNR group; *p* = 0.04); kurtosis (absolute reduction: 1.87 ± 2.19, in pCR group, −0.06 ± 3.78 in pPR/pNR group; *p* = 0.0005); MPP (absolute reduction: 107.91 ± 274.40, in pCR group, −28.33 ± 202.91 in pPR/pNR group; *p* = 0.004)) (Figure 6).

Pre-treatment AUC of entropy for the discrimination between pCR and pPR/pNR was significantly higher than other parameters (AUC entropy = 0.64, IC 95% (0.57–0.71); *p* = 0.0004; AUC kurtosis = 0.56, IC 95% (0.49–0.63); *p* = 0.13; AUC MPP = 0.57, IC 95% (0.50–0.64); *p* = 0.088; AUC skewness = 0.57, IC 95% (0.50–0.64); *p* = 0.11). The optimal pre-treatment entropy cut-off value was ≥6.68, kurtosis cut-off was ≤2.78, MPP cut-off was ≤322.79, and skewness cut-off was >−0.18, and according to these values, the sensitivity and specificity for the detection of pCR for entropy, kurtosis, MPP and skewness were 76.92% and 38.46%, 64.62% and 64.62%, 53.08% and 67.69%, 86.15% and 43.08%, respectively.

**Figure 7:**
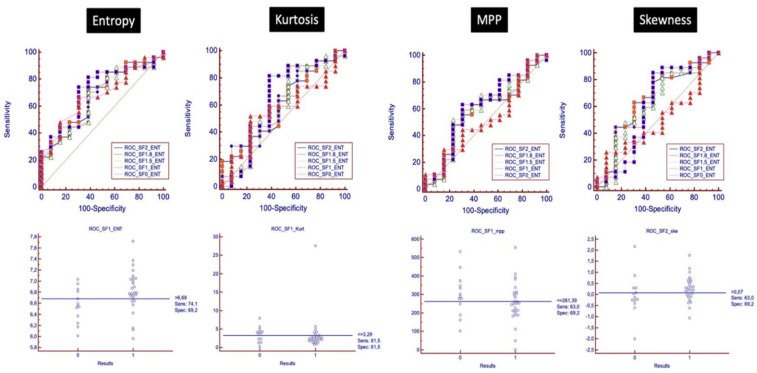
ROC analysis with ROC curves for each filter is shown, analyzing the discriminatory power of baseline texture parameters to distinguish between pCR and pPR/pNR.

Among all filters used, SF1 showed the best diagnostic accuracy to discriminate between pCR and pPR/pNR. The optimal pre-treatment entropy cut-off was ≥6.68, kurtosis cut-off was ≤3.29, MPP cut-off was ≤261.39, and using this value, the sensitivity and specificity for pCR for entropy, kurtosis, MP and skewness were 74.1% and 69.4%, 81.5% and 61.5%, 63% and 69.2%, respectively. The best filter for skewness was SF2, improving sensitivity and specificity up to 63% and 69.2%, respectively (cut-off > −0.07) (Figure 7).

**Figure 8:**
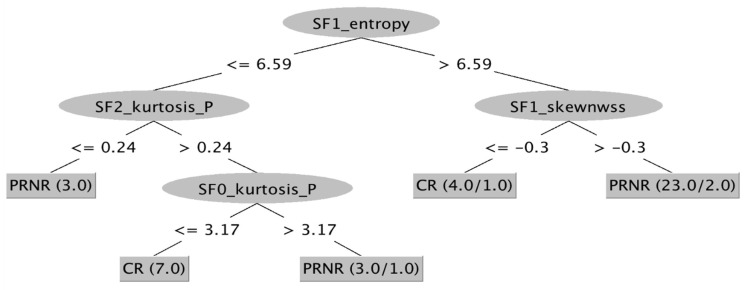
ML-based decisional tree from Weka. All parameters and all filters have been combined to obtain a map able to identify patients who would completely respond to nChRT at the time of the baseline MRI examination. CR: Complete Response; NR: Non-Response; PR: Partial Response; SF: Scale Filter.

Using AI software, a ML-based decisional tree was obtained correlating all TA parameters and filters in order to obtain a model with the best diagnostic performance with the ability to correctly classify all patients who achieved pCR after nChRT during the first MRI examination (Figure 8).

## 4. Discussion

According to our results, TA parameters could be considered accurate imaging biomarkers able to discriminate patients responders to non-responders after nChRT for LARC, representing a potential prognostic factor. In addition, the present study adds value to the existing body of knowledge due to its prospective design, since radiomics and ML studies are often based on retrospectively collected data and thus have a low level of evidence [20].

The mean values of entropy, kurtosis and MPP parameters showed statistically significant differences before and after nChRT in pCR patients, with a decrease in value after nChRT, whereas entropy and skewness demonstrated significant differences before and after nChRT in pPR/pNR patients. Absolute value changes among different TA parameters in pCR and pPR/pNR patients before and after nChRT showed significant differences when entropy, kurtosis and MPP were considered. These TA parameters after nChRT appeared to decrease more in pCR patients compared to pPR/pNR patients. Our results are in line with previous results from Coppola and coworkers [21] and Jalil and colleagues [8], highlighting the potential role of T2-WI-based radiomics in predicting the response to nChRT in LARC. In particular, Jalil and colleagues [8] reported a significant correlation between post-treatment kurtosis and entropy and disease-free survival and recurrence-free survival. Heterogeneity of a tissue is most likely related to oxidative stress and genomic instability that are typical in viable cancerous tissue; thus, the lower values observed in pCR patients allowed us to infer the presence of a less active tissue, such as fibrotic tissue, in accordance with histological complete response [22].

Kurtosis and entropy proved to be the TA parameters with the highest accuracy for pCR prediction (*p* = 0.004). According to De Cecco and colleagues [12,23], kurtosis showed significant results in pCR patients, with a lower value compared to pPR/pNR. Ng and coworkers showed similar results at fine TA parameters, reporting that Kaplan–Meier survival plots for entropy, uniformity, kurtosis, skewness, and standard deviation of the pixel distribution histogram were significantly different for tumors above and below each respective threshold ROC curve optimal cut-off value, with poorer prognosis for ROC optimal values less than 7.89 for entropy and less than 2.48 for kurtosis [22]. This evidence underlines the potential role of kurtosis in discriminating between responders and non-responders prior to nChRT.

Performance of TA evaluated with ROC curves analysis demonstrated entropy as the best parameter to discriminate between pCR and pPR/pNR with a value of 0.64, considering a cut-off ≥ 6.68 (sensitivity and specificity of 76.92% and 38.46%, respectively). This result is in contrast with results reported by De Cecco and colleagues [12,23], who found kurtosis as the parameter with the best AUC. Concordance was found with other studies performed on breast [24] and prostate cancer [25], where T2-WI-derived TA values showed the best AUC in prediction of pCR and tumor aggressiveness, respectively. These results allowed us to infer that entropy is an expression of pixel irregularity and correlates with higher heterogeneity and aggressiveness of tumor. Moreover, entropy is a parameter with good repeatability as shown by Gourtsoyiannis and colleagues [7]; thus, this result has an increased value since it could be adopted in the future as a concrete parameter in clinical practice for patient management.

Among all filters used on images, the best performance of TA parameters with ROC curves discriminating between pCR and pPR/pNR was obtained with SF1 for kurtosis, MPP and entropy, with an increase in AUC values up to 0.71 for entropy (delta: 10.9%), 0.63 for kurtosis (delta: 12.5%), and 0.61 for MPP (delta: 7%). The best filter for skewness was SF2, improving AUC up to 0.65 (delta: 14%).

TA can obtain optional images with filters. In order to obtain altered image pixel intensity patterns and to allow for the extraction of specific structures matching the filter’s width, a Laplacian or Gaussian band-pass filter is commonly used as an advanced image filtration method. Lower filter values coincide with fine texture features, whereas higher filter values outline medium or coarse texture features [26]. Furthermore, this filtration step is designed to wipe off noise and enhance edges, making measurements less susceptible to technical differences. Denoising or gray-level standardization steps have been employed as a pre-measurement procedure in order to reduce technical differences rather than biologic ones, and to improve reproducibility. In our study, the introduction of an SF1 filter resulted in an improved diagnostic accuracy for most of the parameters, reducing noise and enhancing image features related to the biological tissues.

Unfortunately, the application of filters entails that the datasets enlarge considerably, resulting in difficulties to extract summary data and to compute results without any error. In such cases, software based on AI algorithms could come to aid. In our study, a ML-based approach allowed us to build a decisional map according to which the most relevant TA parameters and filters were combined: this map should be intended as a decisional tree, able to detect patients who would completely respond to nChRT.

Despite the encouraging results, our study has some limitations. First, the sample size of patients analyzed was limited. Therefore, observed correlations among TA parameters, response to therapy and histologic results should be confirmed in larger studies. Second, we did not correlate T2-WI TA with other imaging biomarkers that have been reported to be predictors of response to nChRT, including diffusion-weighted imaging (DWI), with apparent diffusion coefficient (ADC), and dynamic contrast-enhanced MRI (DCE–MRI) parameters. Hence, we could not compare its predictive power with other imaging biomarkers. Third, the absence of standardized protocol for TA, such as selection of the filtration; more studies are needed to identify which protocol is more accurate. Fourth, no follow-up was available at the moment of the analysis, with the lack of results regarding the predictive value of tumor heterogeneity based on patient survival. In the future, a longitudinal study would be helpful in the assessment of the prognostic value of TA in rectal cancer. Fifth, the TA software we had the possibility to use at our Institution is currently not compliant with the image biomarker standardization initiative (IBSI) benchmarks. Moreover, post-treatment MRI examination was performed at the end of nChRT, with a possible early clinical evaluation compared to the surgical resection with histopathological assessment (6–8 weeks after). This could have led to a bias in terms of clinical–pathological correlation; however, an individualized multidisciplinary risk assessment was carried out for each patient in order to develop or refine the disease management plan and the accurate time to surgery after nChRT, mitigating this issue as much as possible. Finally, the decisional tree obtained from AI software has a perfect diagnostic accuracy only if applied in a population identical to our study population. It is necessary to validate the map through a larger and different sample to test its real clinical value. However, we applied the decisional tree to a sub-group of patients extracted from our population, obtaining promising results in terms of diagnostic accuracy.

In conclusion, our preliminary results infer that TA parameters obtained from T2-WI MRI images can potentially have an important role in patient clinical management as imaging biomarkers of tumoral response to nChRT, in particular stratifying patients with pCR and those with pPR or pNR at the time of the baseline MRI examination.

## Figures and Tables

**Table 1:** Demographic and clinical data of patients included in the study.

Characteristic	All Participants (*n* = *40*)	pCR (*n* = *13*)	pPR (*n* = *22*)	pNR (*n* = *5*)	*p* Value
*Sex*					0.62
Male	24 (60%)	8 (62%)	14 (64%)	2 (40%)	
Female	16 (40%)	5 (38%)	8 (36%)	3 (60%)	
Age, years *	64 ± 9 (39–82)	57 ± 10 (39–74)	65 ± 11 (43–82)	64 ± 10 (52–80)	0.08
*Tumor Grade*					0.18
G1	15 (38%)	8 (62%)	6 (27%)	1 (20%)	
G2	18 (45%)	4 (31%)	12 (55%)	2 (40%)	
G3	7 (17%)	1 (7%)	4 (18%)	2 (40%)	
*T—Stage*					0.56
T1	11 (28%)	6 (46%)	4 (18%)	1 (20%)	
T2	13 (33%)	4 (31%)	8 (36%)	1 (20%)	
T3	9 (22%)	2 (15%)	5 (23%)	2 (40%)	
T4	7 (18%)	1 (8%)	5 (23%)	1 (20%)	
*N—Stage*					0.17
N0	22 (55%)	10 (77%)	11 (50%)	1 (20%)	
N1	12 (30%)	2 (15%)	8 (36%)	2 (40%)	
N2	6 (15%)	1 (8%)	3 (14%)	2 (40%)	
*TRG*					/
0	4 (10%)	/	/	4 (80%)	
1	1 (2%)	/	/	1 (20%)	
2	19 (47%)	/	19 (86%)	/	
3	3 (8%)	/	3 (4%)	/	
4	13 (33%)	13 (100%)	/	/	

Unless otherwise indicated, data are numbers with percentages in parentheses. * Data are means ± standard deviations, with ranges in parentheses. pCR: Pathological Complete Response; pPR: Pathological Partial Response; pNR: Pathological Non-Response; TRG: Tumor Regression Grade.

## Data Availability

Data were collected and archived at Electronic Archive of Sapienza University of Rome.

## Abbreviations

AI	Artificial Intelligence
AUC	Area Under the ROC Curve
CT	Computed Tomography
LARC	Locally Advanced Rectal Cancer
ML	Machine Learning
MPP	Mean of Positive Pixels
MRI	Magnetic Resonance Imaging
nChRT	Neoadjuvant Chemoradiotherapy
pCR	Pathological Complete Response
pNR	Pathological Non-Response
pPR	Pathological Partial Response
ROC	Receiver Operating Characteristic Curve
ROI	Region of Interest
SF	Scale Filter
TA	Texture Analysis
TME	Total Mesorectal Excision
TRG	Tumor Regression Grade
